# Pearls and Pitfalls of Phrenic Nerve Transfer for Shoulder Reconstruction in Brachial Plexus Injury

**DOI:** 10.1055/s-0041-1722979

**Published:** 2021-02-10

**Authors:** Kazuteru Doi, Sei Haw Sem, Bipin Ghanghurde, Yasunori Hattori, Sotetsu Sakamoto

**Affiliations:** 1 Department of Orthopedic Surgery, Ogori Daiichi General Hospital, Yamaguchi City, Yamaguchi Prefecture, Japan; 2 Department of Orthopaedics, Hospital Kuala Lumpur, Kuala Lumpur, Malaysia; 3 Hand surgery, Kusum Orthopedic Centre, Mumbai, Maharashtra, India; 4 Hand surgery, Wadia Hospital, Mumbai, Maharashtra, India; 5 Hand surgery, Surya Hospital, Mumbai, Maharashtra, India

**Keywords:** brachial plexus injury, phrenic nerve transfer, suprascapular nerve

## Abstract

**Objectives** The purpose of this study was to report the functional outcomes of phrenic nerve transfer (PNT) to suprascapular nerve (SSN) for shoulder reconstruction in brachial plexus injury (BPI) patients with total and C5–8 palsies, and its pulmonary complications.

**Methods** Forty-four out of 127 BPI patients with total and C5–8 palsies who underwent PNT to SSN for shoulder reconstruction were evaluated for functional outcomes in comparison with other types of nerve transfers. Their pulmonary function was analyzed using vital capacity in the percentage of predicted value and Hugh-Jones (HJ) breathless classification. The predisposing factors to develop pulmonary complications in those patients were examined as well.

**Results** PNT to SSN provided a better shoulder range of motion significantly as compared with nerve transfer from C5 root and contralateral C7. The results between PNT and spinal accessory nerve transfer to SSN were comparable in all directions of shoulder motions. There were no significant respiratory symptoms in majority of the patients including six patients who were classified into grade 2 HJ breathlessness grading. Two predisposing factors for poorer pulmonary performance were identified, which were age and body mass index, with cut-off values of younger than 32 years old and less than 23, respectively.

**Conclusions** PNT to SSN can be a reliable reconstructive procedure in restoration of shoulder function in BPI patients with total or C5–8 palsy. The postoperative pulmonary complications can be prevented with vigilant patient selection.

## Introduction

Brachial plexus injury (BPI) is a debilitating injury that causes significant disabilities especially in patients with total or C5–8 type of palsies, where the treatment option is limited, and rehabilitation program is challenging. 1

However, it is possible to restore a good functional upper limb with the advancement of surgical techniques in BPI. This involves multiple reconstructive procedures of nerve transfer (NT) with or without free muscle transfer (FMT). 2

Traditionally, the spinal accessory nerve (SAN) and intercostal nerve (ICN) are used as donor nerves in shoulder and elbow reconstruction for BPI patients. Additional donor nerves, e.g., contralateral C7 (CC7) root and phrenic nerve (PN), were introduced 1 3 to accommodate the increased number of reconstructive procedures to restore prehensile function of a flail upper limb by FMT.

We used to prefer using CC7 as a donor nerve in our center, but the outcomes were unsatisfactory. Therefore, we decided to use PN as a donor nerve for shoulder or elbow reconstruction in BPI for selected cases. PN is a powerful motor nerve that is able to produce consistent good results in NT surgery especially for restoration of elbow flexion.

The most cited argument against the use of PN as a donor nerve is the short- and long-term deleterious respiratory effects secondary to diaphragmatic paralysis. 4 5 Generally, phrenic NT (PNT) is preferred by the Asian surgeons, 6 7 8 9 whereas it is less favorable among the American-European surgeons, maybe due to the differences in the patients' sociodemographic background. 1

The objectives of this study were to compare the functional outcomes of PNT with other surgical procedures and to analyze its effects on the pulmonary function.

## Materials and Methods

This was a retrospective study conducted in a single institution involving BPI patients with total and C5–8 type of palsies 10 who underwent NT to suprascapular nerve (SSN) by a single senior surgeon between October 2001 and December 2016. This study was approved by the local hospital institutional review board.

**Fig. 1:**
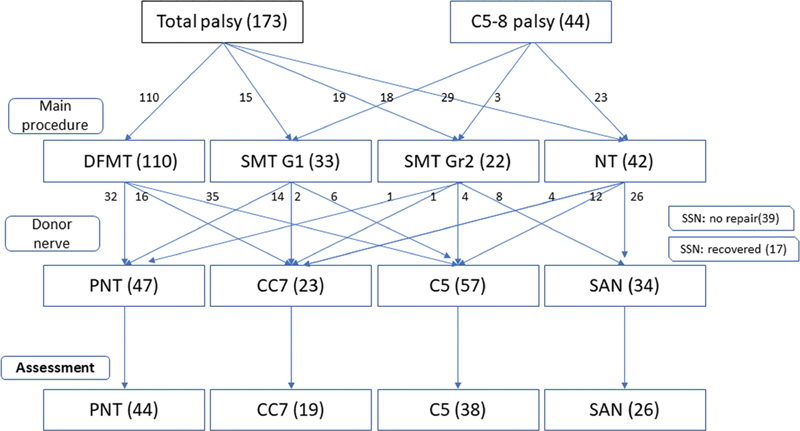
Algorithms for patient selection and treatment by associated injury in total and C5–8 types of palsy and surgical treatment protocol. C5, C5 nerve root transfer; CC7, contralateral C7 nerve root transfer; DFMT, double free muscle transfer; FFMT, functioning free muscle transfer; G1, gracilis 1 of stage I operation in DFMT; G2, gracilis 2 of stage II in DFMT; NT, nerve transfer; PNT, phrenic nerve transfer; SAN, spinal accessory nerve; ScA, subclavian artery; SMT, single muscle transfer; SSN, suprascapular nerve.

A total of 127 out of 217 patients with no functioning muscles for shoulder motion fulfilled the inclusion criteria. The exclusion criteria were spontaneous recovery of SSN, short follow-up duration, loss of follow-up, associated SAN or spinal cord injury, postoperative vascular complications of FMT, and additional secondary procedures. The algorithm for patient selection and treatment is illustrated in Fig. 1 . Patients were stratified into four different groups according to the donor nerves for shoulder reconstruction (PN, CC7, C5, and SAN) for further analysis.

In our center, we prioritized the elbow reconstruction followed by restoration of shoulder and finger motion. NT surgery was only performed for patients with early presentation (within 6 months after the injury).

### Total Palsy

The surgical treatment options for patients with total palsy were NT and/or FMT, either single FMT (SMT) or double FMT (DFMT). 11 The treatment for each patient was chosen based on the time from injury, availability of donor nerves and recipient vessels, and patient's desire.

### C5–8 Palsy

There were two surgical treatment options for elbow flexion and finger extension restoration in addition to shoulder reconstruction for patients with C5–8 palsy, which were FMT or ICN to musculocutaneous nerve (MCN) transfer followed by secondary tendon transfer for thumb and finger extension using palmaris longus and flexor digitorum superficialis. The treatment of choice was depending on the availability of PN, SAN, and subclavian artery (ScA).

### Double Free Muscle Transfer

SAN and two ICNs had to be used as donor nerves imperatively in patients who required DFMT. Therefore, the available options of donor nerve for transfer to SSN were C5 nerve root, CC7 nerve root, and PN. 1 2 12 Among these three donor nerves, C5 nerve root grafting was the best option if it was uninjured. We used to perform CC7 root transfer to SSN with vascularized ulnar nerve graft before year 2006, but it was abandoned due to unsatisfactory outcomes. Since then, PNT to SSN was the treatment of choice if C5 root was not available.

### Single Free Muscle Transfer

We performed SMT for patients with associated ScA or SAN injury. For this group of patients, they underwent NT for shoulder reconstruction and SMT for elbow flexion and finger flexion or extension reconstruction. SMT can be either one of the two types: gracilis one (Gr1) and gracilis two (Gr2), which were innervated from SAN and ICNs, respectively. Gracilis was harvested together with a skin paddle for monitoring purpose.

### Nerve Transfer

This conventional surgical technique was performed for patients with early presentation (<6 months after injury), refused FMT due to personal or socioeconomic reasons, and associated ScA and/or SAN injury resulted in insufficient recipient vessels and donor nerves.

#### Selection of Donor Nerve for Shoulder Reconstruction

The donor nerves are limited in surgical reconstruction for BPI patients and the choice of donor nerve for shoulder reconstruction depends on the types of reconstruction for elbow and fingers.

Our first choice of donor nerve for shoulder reconstruction would be the C5 nerve root, which was transferred to SSN via sural nerve graft. The availability of C5 root was determined by magnetic resonance imaging (MRI) preoperatively and further confirmed by electrical stimulation intraoperatively. 13

In patients with C5 root avulsion, the second choice of donor nerve for SSN would be CC7 or PN in patients for FMT and SAN in patients for NT (ICN to MCN transfer for elbow flexion).

The contraindications for PNT were history of lung contusion, multiple rib fracture, and poor pulmonary function test (PFT). The possibility of PN palsy was determined by dynamic chest radiographs preoperatively. 13

#### Surgical Procedures

*Shoulder reconstruction* : We performed NT to SSN and exploration of the C5 nerve roots through two separate transverse cervical and supraclavicular incisions for all the patients. The availability of C5 root was determined by direct electrical nerve stimulation. The nerve root was deemed as intact if the serratus anterior muscle was contracted upon electrical stimulation and it would be used to connect to the SSN via sural nerve graft. If the C5 root was avulsed, then NT would be proceeded with other choice of donor nerves as described above. For the CC7 nerve root as the donor nerve, the posterior division of the CC7 was transected over the contralateral neck, tunneled through the upper chest region subcutaneously, and connected to SSN with a vascularized ulnar nerve graft. As for PNT, the PN was identified and harvested distally underneath the sternal attachment of sternocleidomastoid muscle for direct coaptation to the SSN. For SAN to SSN transfer, the SAN was transected just distal to the branch to middle trapezius to allow direct coaptation with SSN by using the anterior approach.

*Elbow and finger reconstruction* : DFMT involved two-stage surgeries with stage I for NT to restore shoulder function as described together with Gr1 FMT for elbow flexion and finger extension, which was innervated by SAN. 2 Stage II procedures involved Gr2 FMT with innervation from sixth and seventh ICNs for finger flexion together with NT of third to fifth ICNs to triceps muscle branch of radial nerve for elbow extension.

As for SMT, it could be either Gr1 or Gr2 depending on the availability of SAN. The NT performed for elbow flexion was the conventional third to fifth ICNs to MCN transfer ( Fig. 1 ).

The outcomes were analyzed in several aspects:

- Early outcome was evaluated based on the reinnervation timing of the infraspinatus muscle. This was performed with needle electromyography (EMG) at monthly interval, which was started at 2 months post-NT.
- Functional outcome of the patients: the range of motions of the shoulder and elbow joints was measured with a goniometer and the power of the elbow flexion was recorded with a stationary dynamometer, the Kin-Com (Chattecx, Chattanooga, Tennessee, United States). Besides that, the Disabilities of the Arm, Shoulder and Hand (DASH) questionnaire was used to assess the patient's quality of life.
- Mid-term outcome was assessed based on the lung function postoperatively. The PFT was performed with a plethysmograph and results of vital capacity in the percentage of predicted value (%VC) and forced expiratory volume in 1 second (FEV _1_ ) were collected and analyzed. Indices of spirometry are varied according to the age, sex, height, and ethnicity; thus, the result has to be compared with the adjusted reference values. %VC is used to determine the severity of respiratory muscle involvement in neuromuscular diseases including diaphragm dysfunction 14 with a standard cut-off value of 80%. A result of <79% is suggestive of restrictive lung diseases such as pulmonary contusion, atelectasis, PN palsy, and trauma to the chest wall. Patients with unilateral diaphragmatic paralysis usually have %VC of more than 70% and largely asymptomatic without exercise limitation and only detected on incidental radiographic findings. On the other hand, patients with bilateral diaphragmatic paralysis will have symptoms of severe respiratory failure and dyspnea at rest with %VC of less than 50%. 14

FEV _1_ is used to assess the severity of obstructive pulmonary diseases such as asthma, emphysema, and chronic bronchitis. The normal cut-off value of FEV _1_ is 70%.

- The correlation between pulmonary function and predisposing factors: PFTs of all patients were performed by a single technician using a pulmonary function meter (MasterScreen Diffusion, Jaeger) in the sitting position. The results of VC and FEV _1_ were collected, analyzed, and standardized into percentage of reference values from a healthy national population [% VC and FEV _1_ (%)] to eliminate the bias of age, sex, height, and weight. The PFTs were performed once before surgeries (PNT) and four times after the surgeries (PNT and ICN transfer) at regular intervals.

The patients' postoperative respiratory efforts were classified according to the Hugh-Jones (HJ) classification, 15 which was based on the daily activities affected. The possible predisposing factors for poorer postoperative respiratory effort such as age, body mass index (BMI), and %VC were examined. A receiver operating characteristic (ROC) curve was used to analyze and determine the cut-off point between HJ-1 and HJ-2.

## Statistical Analysis

All data are presented as means and ranges. Based on the data normality, either Student's *t* -test or Mann–Whitney U-test was used for comparison between two independent levels, and one-way analysis of variance or Kruskal–Wallis test was used for comparison of more than two independent levels. The tests that were used for multiple comparisons were Dunnett's test or Steel–Dwass test.

A ROC curve was used to determine the optimal cut-off point between HJ grade 1 and grade 2 with the area under the curve (AUC) as the diagnostic performance. A test with AUC of 0.9 to 1.0 has high accuracy, while 0.7 to 0.9 is moderate accuracy, and 0.5 to 0.7 indicates low accuracy. The level of significance was set at 0.05 ( *p* < 0.05).

## Results

There were no serious systemic complications documented. Ten gracilis grafts in DFMT developed acute vascular complications postoperatively, but all were managed to salvage and reanastomose successfully.

A total of 127 patients were reviewed with the minimum follow-up period of 18 months after last surgery. The overall mean age of patients was 28.3 years old (range: 11–62). The mean BMI was 23.0 (15.5–42.2). Majority of the patients underwent PNT (44 patients) followed by C5 root transfer (38 patients), SAN transfer (26 patients), and CC7 transfer (19 patients) to SSN. Although there were significant differences ( *p* = 0.036) for the mean age between the four groups, the effect size was small clinically. The mean BMI was comparable among the four groups. The time of infraspinatus muscle reinnervation as demonstrated by EMG was faster in PN and SAN transfer groups as compared with the patients with CC7 and C5 transfer. The durations of follow-up were comparable in all four groups ( Table 1 ).

**Table 1:** Demographics of donor nerves transferred to suprascapular nerve for shoulder reconstruction

Donor nerve		PN	CC7	C5	SAN		
*n*		44	19	38	26	*p*	ES (unit)
Mean age (y)		29	24	26	34	**0.036**	3.270
Gender	Male	43	17	34	24	0.460	
	Female	1	2	4	2		
Mean BMI		23	23	22	24	0.440	0.473
Mean time to EMG reinnervation (mo)		4	7	7	4	**<0.0001**	1.297
Mean follow-up period (mo)		34	33	38	41	0.756	3.156

Abbreviations: BMI, body-mass-index; C5, cervical 5 nerve root; CC7, contralateral cervical 7 nerve root; PN, phrenic nerve; SAN, spinal accessory nerve.

Note: Values in bold indicate statistical significance. The level of significance was set at *p* < 0.05.

## Comparison of Shoulder and Elbow Functions

**Fig. 2:**
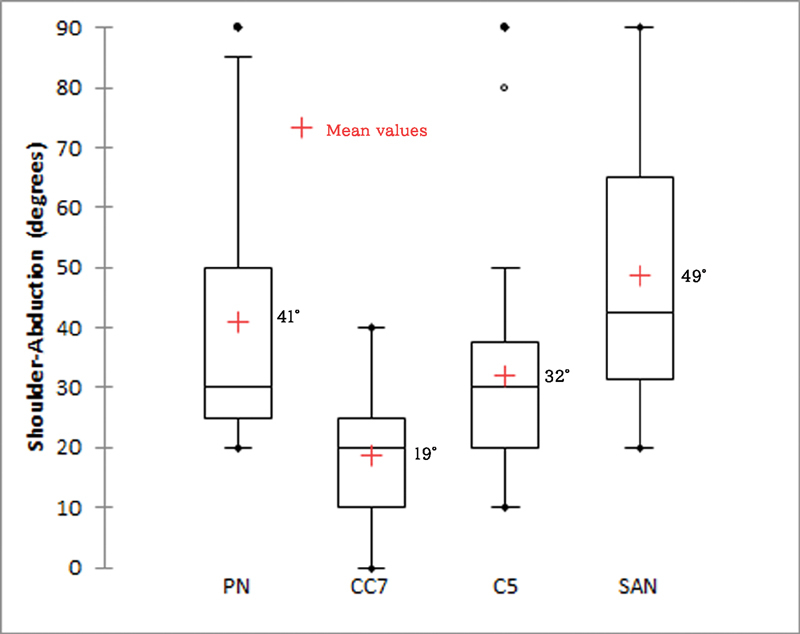
Comparison of postoperative range of shoulder abduction between four donor nerves. (Kruskal–Wallis test, *p* < 0.0001).

In comparison of shoulder abduction, flexion, and rotation arc between all four groups, patients with PN and SAN transfer regained a better range of motions as compared with CC7 and C5 transfer groups. The results of PN and SAN groups were comparable in all directions of shoulder motions and patients with CC7 transfer had the worst performance ( Figs. 2 3 4 , Table 2 ). The median total active motion of the fingers after DFMT was 40 degrees (interquartile range: 29–60 degrees). Intercostal-brachial to median NT was performed for hand sensory restoration, and 21 and 44% of the patients achieved S2 and S1 fingertip sensibility, respectively, according to the British Medical Society Grading.

**Fig. 3:**
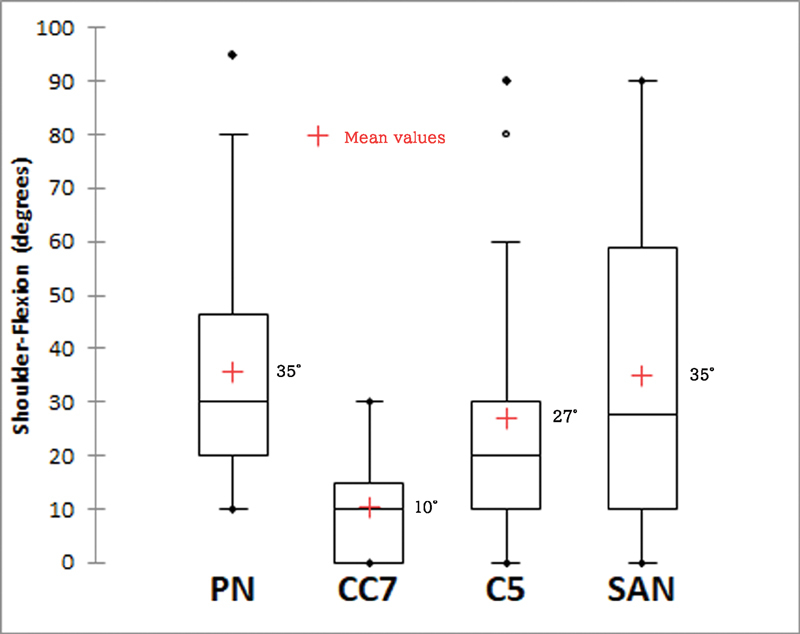
Comparison of postoperative range of shoulder flexion between four donor nerves. (Kruskal–Wallis test, *p* < 0.0001).

**Fig. 4:**
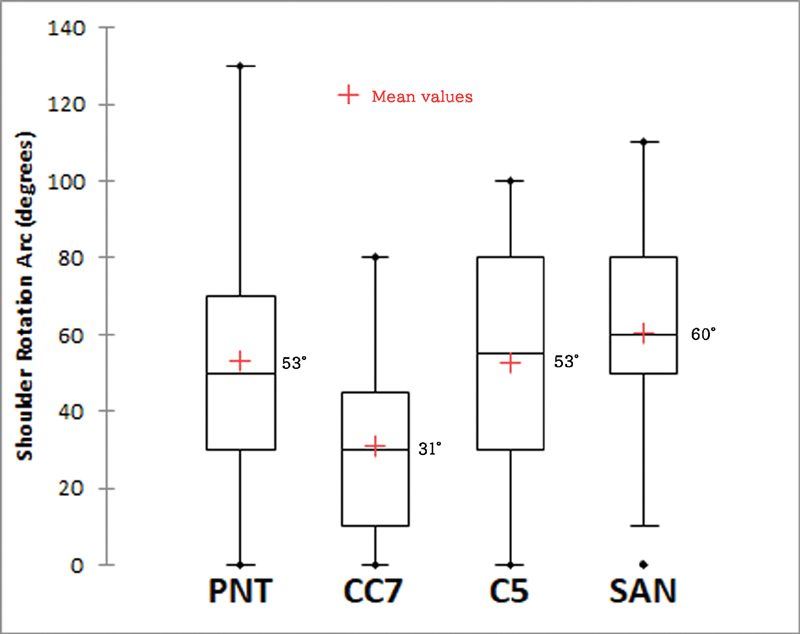
Comparison of postoperative range of shoulder-rotation arc between four donor nerves. (Kruskal–Wallis test, *p* = 0.01).

**Table 2:** Comparison of functional and quality-of-life recovery between PN, CC7, C5, and SAN transfer to SSN

Function and quality-of-life recovery			Donor nerve				
Item		Detail	PN	CC7	C5	SAN	*p*
Mean shoulder ROM (degrees)		Abduction	41	19	32	49	**<0.0001**
		Flexion	35	10	27	35	**<0.0001**
		Rotation arc	53	31	53	60	**0.01**
Mean elbow flexion	ROM	Degrees	112	117	114	112	0.646
	Power by Kin-Com	Concentric (%) a	15	11	18	11	**0.023**
		Eccentric (%) a	16	14	19	13	0.165
Mean DASH disability score		Preoperative	50	59	63	59	**0.011**
		Postoperative	38	41	36	42	0.529
		Improvement	12	18	30	17	0.068

Abbreviations: DASH, Disabilities of the Arm, Shoulder and Hand; ROM, range of motion.

Note: Values in bold indicate statistical significance. The level of significance was set at *p* < 0.05.

a Percent of uninvolved side.

The mean elbow flexion in degrees and power measured by a Kin-Com dynamometer were comparable in all four groups but the measured concentric contraction of elbow flexion in the C5 transfer group was significantly higher than those with SAN transfer ( Table 2 ).

Preoperative, postoperative, and improvement of the DASH scores were comparable in all four groups but there was a significant difference between PN and C5 transfer groups in terms of the preoperative DASH score.

## Pulmonary Function in Patients with PNT

**Fig. 5:**
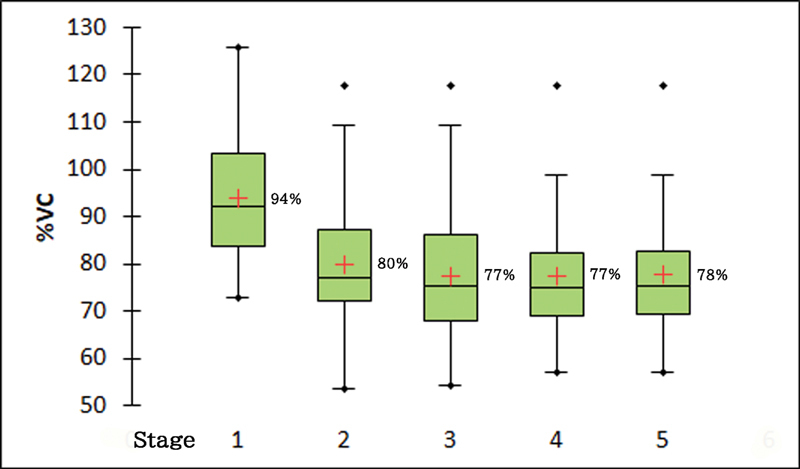
Postoperative progress of pulmonary function test by percent vital capacity (%VC) of patients with phrenic nerve transfer (PNT). Stage 1 (before PNT); 2 (2 months after PNT); 3 (5 months after PNT); 4 (8 months after PNT); 5 (32 months after PNT). Intercostal nerve transfer has been done just before stage 3.

Forty-two out of 44 patients who underwent PNT completed the PFTs and were subjected for further analysis. There was significant reduction in %VC of the patients between before and after PNT with an average of 14% ( *p* < 0.0001) reduction, but no further reduction of the %VC after ICN transfer was noted and it remained static until the last examination at an average of 32 months after PNT ( Fig. 5 ).

**Fig. 6:**
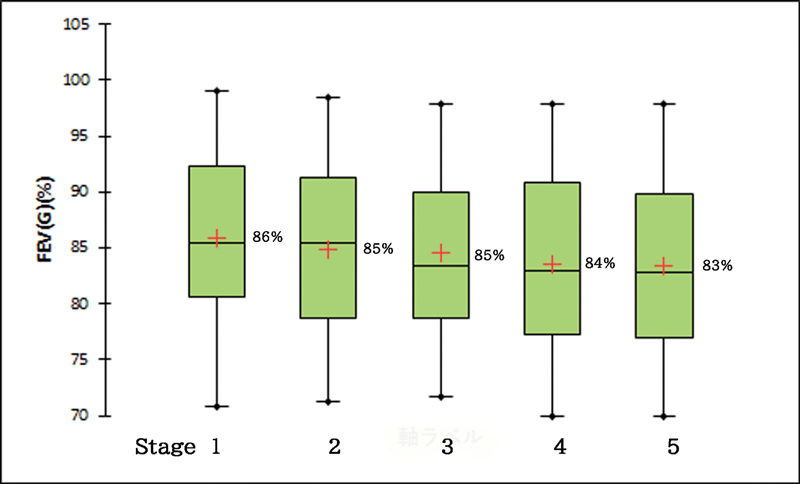
Postoperative progress of pulmonary function test by forced expiratory volume in 1 second [FEV _1_ (%)] of patients with phrenic nerve transfer (PNT). Stages are same as Figure 5 .

As for FEV _1_ (%), no significant differences were noted in patients before and after PNT ( *p* = 0.771). All the measured values were within normal limits (> 70%; Fig. 6 ).

Upon assessment of the 42 patients based on HJ classification, 15 36 patients were classified as grade 1 (HJ-1), 6 patients as grade 2 (HJ-2), and no patient as grade 3, 4, or 5. All patients were satisfied with their performances and no further treatment was required.

Further analysis of the patients between HJ-1 and HJ-2 revealed significant differences in terms of the patients' age and BMI, where the patients with HJ-2 were older with higher BMI ( Table 3 ). No significant difference was found between the two groups for the preoperative %VC and FEV _1_ (%). Postoperatively, the patients in HJ-2 had lower %VC and FEV _1_ (%) than patients in HJ-1 with a higher amount of reduction, and the differences were significant statistically.

**Table 3:** Comparison of %VC and FEV _1_ (%) according to Hugh-Jones classification

Hugh-Jones grading		HJ-1	HJ-2	
*N*		36 a	6 a	*p*
Mean age		29	43	**0.008**
Mean BMI		22	27	**0.012**
Mean follow-up periods (mo)		32	31	0.913
Mean %VC	Preoperative	93	95	0.824
	Postoperative	79	69	**0.025**
	Reduction	15	26	**0.014**
Mean FEV1%	Preoperative	86	81	0.119
	Postoperative	85	76	**0.006**
	Reduction	1	6	**0.047**

Abbreviation: BMI, body mass index; FEV _1_ (%), forced expiratory volume in 1 second; HJ, Hugh-Jones breathlessness classification; %VC, percent vital capacity.

Note: Values in bold indicate statistical significance. The level of significance was set at *p* < 0.05.

a Only patients who completed preoperative and four postoperative pulmonary function test examinations. Two patients excluded.

**Fig. 7:**
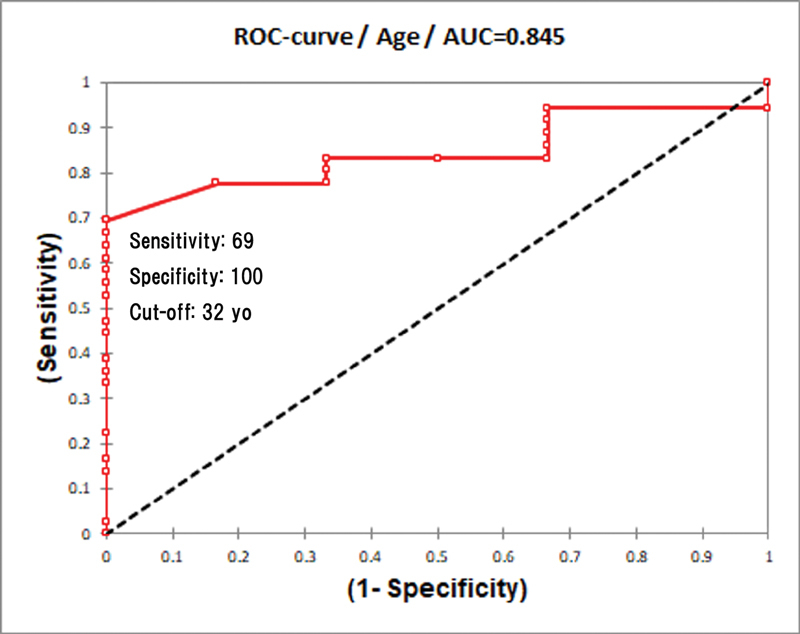
Receiver operating characteristic (ROC) curve of age between Hugh-Jones 1 and 2 grading. AUC, area under a ROC curve: yo, years old.

**Fig. 8:**
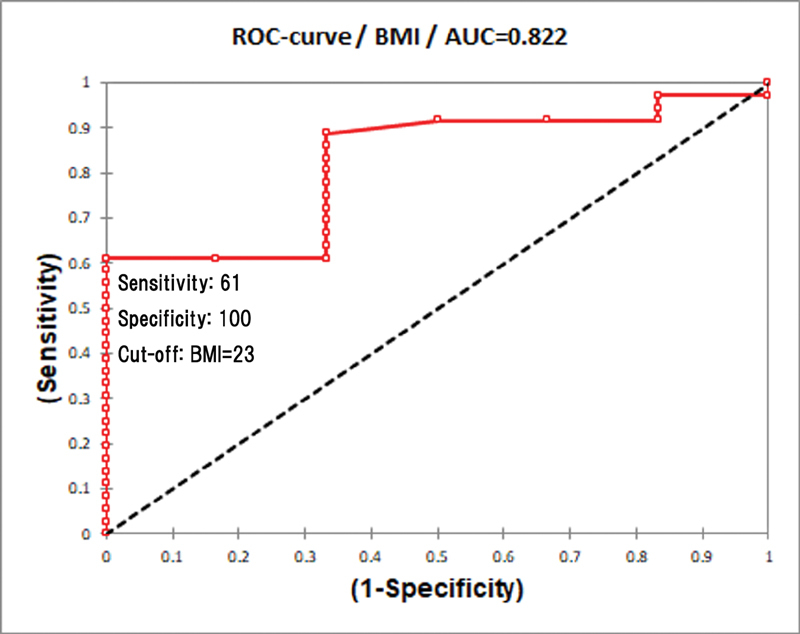
Receiver operating characteristic (ROC) curve of BMI between Hugh-Jones 1 and 2 grading. AUC, area under a ROC curve; BMI, body mass index.

On ROC curve analysis, the cut-off value for patients' age between HJ-1 and 2 was 32 with a sensitivity of 69% and a specificity of 100% ( Fig. 7 ). As for patients' BMI, the cut-off point was 23 with a sensitivity of 61% and a specificity of 100% ( Fig. 8 ). The AUCs for patients' age and BMI were 0.845 and 0.822, respectively ( Figs. 2 and 3 ).

The preoperative %VC had a very low AUC (0.447) to be analyzed with low accuracy. However, the postoperative %VC and %VC reduction showed higher AUCs of 0.792 and 0.824, respectively. The cut-off value of postoperative %VC was 71%, whereas 25% of %VC reduction was the cut-off point between HJ-1 and HJ-2. Both the postoperative %VC and %VC reduction had a sensitivity of 81% and a specificity of 83%.

## Discussion

Restoration of prehensile hand function together with shoulder and elbow reconstructions is possible nowadays in BPI patients with total palsy following the introduction of FMT. However, more donor motor nerves are required to achieve the purpose. PN is one of the useful donor nerves and has been used for reconstruction of the elbow flexion or shoulder abduction. 6 7 8 9

Satisfactory outcomes of shoulder function in terms of ROM and power grading using the Medical Research Council scale have been reported in the literature for BPI patients with varied types of palsy and accompanied surgical procedures. 6 7 8 9 In this study, we attempted to eliminate the influence of supplementary muscles on the shoulder function by analyzing the outcomes of PNT in shoulder reconstruction for BPI patients with total and C5–8 palsies only, of whom the serratus anterior muscle was not functioning, and we only performed NT to SSN but not axillary nerve.

From our patient series with NT to SSN, poorer outcomes were noted in patients with DFMT as compared with those with NT only. This is due to the strong adduction and internal rotation forces acted by the transferred muscles, of which there was reduction of the improvement of shoulder abduction and external rotation by SSN repair. 11 12 Comparison among the four NTs to SSN revealed that both PNT and SAN transfer provided equally better outcomes in terms of shoulder motions because of the shorter time of reinnervation to the target muscles. 12 16 17 18

C5 nerve root transfer provided unexpected poor results despite the short distance from the donor nerve to the recipient nerve. This could be due to lack of healthy motor nerve fibers in the proximal C5 stump even with the positive findings from MRI preoperatively and electrical stimulation intraoperatively. The poorest result among the four NTs was from CC7 transfer, with achieving only stabilization of the glenohumeral joint without a confirmable range of shoulder motions. This poor outcome was mainly due to the difficulty of cortical reorganization (neuroplasticity), where the control center for the receptor muscle is located in the other cerebral hemisphere.

In our center, the first choice of donor nerve for shoulder reconstruction in patients for DFMT was the C5 nerve root, of which the SAN would be used as a donor nerve for FMT. In cases where the C5 nerve root was avulsed (preganglionic injury), the next choice of donor nerve would be PN instead of CC7. There are several advantages of PNT in comparison with other NT for SSN repair, such as a long PN can be harvested for direct coaptation with SSN without nerve grafting, it has a large diameter with single nerve fascicle to facilitate coaptation, and faster time of reinnervation to infraspinatus muscle.

The efficacy of PNT and its long-term safety have been reported in the literature. 4 19 20 21 However, there are still widespread concerns about the effect of PNT on respiratory function especially among American and European surgeons, and the need of further confirmatory reports before adoption of this surgical technique has been suggested. 22 23 24

We attempted to delineate the predisposing factors for worsening respiratory function post-PNT in our patient series with pre- and postoperative respiratory assessments. The %VC reduced significantly after PNT, and it reduced further after ICN transfer, but the reduction was minimal and not significant statistically ( Fig. 5 ). The reduced %VC did not recover even after 3 years postoperatively.

Comparison between patients in groups HJ-1 and HJ-2 revealed that the difference in %VC reduction was significant statistically ( Table 2 ). Further analysis showed that the cut-off values of postoperative %VC and % VC reduction between HJ-1 and HJ-2 were 71 and 25%, respectively. However, these were postoperative values and they were less useful in the clinical setting preoperatively.

Two predisposing factors for poorer postoperative respiratory function were identified, which were patient's age and BMI. Those who were younger than 32 years and BMI less than 23 did not develop any respiratory symptoms postoperatively. As for preoperative %VC, it was less sensitive and specific as an indicator to predict postoperative respiratory function. However, a value of more than 90% was preferable.

There were several limitations in our study. A relatively short follow-up period in this study might not have captured the decreasing trend of %VC over the years as pointed out by several authors. 25 26 A longer follow-up duration could increase the strength of evidence but it would be difficult to follow up the patients over decades at the present time. The association between BMI and health risks could differ according to populations and ethnic groups; therefore, the BMI cut-off value in our study might not be applicable to the Europeans or Americans.

Most of the patients developed eventration of diaphragm after PNT but majority of them were asymptomatic. However, morbidly obese patients tend to have symptomatic diaphragmatic eventration. 26 It is important to educate the patients to stop smoking and reduce or maintain body weight to prevent pulmonary complications.

This study showed that all of the patients had reduced %VC after PNT. No patient developed severe pulmonary complications despite the reduction of %VC as long as it did not fall below the critical level. Several important factors to consider before PNT include patient's age, BMI, preoperative %VC, and the risk-to-benefit ratio in restoration of arm function in comparison with other conventional procedures. Furthermore, the contraindications for PNT must be respected to ensure its safety, such as age less than 3 years old, obesity, and history of severe thoracic trauma or cardiopulmonary diseases. 5

In conclusion, PNT is a safe and reliable surgical reconstructive procedure to restore shoulder function in BPI patients with total and C5–8 palsies, especially in young patients (<32 years old) with low BMI (<23) and good preoperative %VC (> 90%) with postoperative regular health care.
